# Assessment of Phenotype, Oxidative Stress and Biomacromolecule Oxidation in Human Chronic Skin Wound Epidermis in Active Debridement Tissue Versus Endstage Wounds

**DOI:** 10.1111/wrr.70196

**Published:** 2026-09-02

**Authors:** Dylan Tinney, Justin Carmichael, Punkuj Chawla, David Keast, Douglas W. Hamilton

**Affiliations:** ^1^ Department of Anatomy and Cell Biology, Schulich School of Medicine and Dentistry Western University London Ontario Canada; ^2^ Chronic Wound and Lymphedema Clinic, St. Joseph's Healthcare: Parkwood Institute London Ontario Canada; ^3^ Dentistry, Schulich School of Medicine and Dentistry Western University London Ontario Canada

**Keywords:** cytokeratin (CK), keratinocyte activation, oxidative stress, re‐epithelialization, skin healing, wound chronicity

## Abstract

Impairment of cutaneous healing occurs in association with numerous comorbidities including diabetes, vascular insufficiency and immobility. Standard management of chronic wounds includes removal of necrotic tissue and infection management, but no biomarkers are used to guide appropriate debridement distance. We hypothesized that by understanding the spatial distribution of proteins associated with keratinocyte differentiation and activation, oxidative stress biomarkers, as well as accumulation of oxidative damage, debridement protocols could be refined. Human tissue was isolated from (1) routine debridement procedures or (2) endstage at limb amputation. In debridement samples, wound edge epithelium typically exhibited hyperproliferation with or without epibole, with endstage wounds exhibiting a much thinner epidermis. Cytokeratin 14 expression was increased throughout the suprabasal epidermis with cytokeratin 10 and filaggrin expression attenuated; endstage wounds displayed downregulation of both cytokeratin 10 and filaggrin further from the wound edge than observed in debridement tissue. Cytokeratin 6, 16 and 17 were expressed in the wound edge and adjacent uninjured epidermis in both debridement and endstage wounds, while cytokeratin 16 and 17 were expressed in the non‐involved interfollicular epidermis in endstage limbs. Both phospho‐nuclear factor kappa B and the antioxidant transcription factor phospho‐nuclear factor‐erythroid 2‐related factor 2 show increased nuclear localization in the keratinocytes of endstage compared to debrided wounds concomitant with increased oxidative damage to lipids, protein and DNA in the epidermis. Our observations demonstrate increased tissue damage in endstage wounds compared to debridement tissue, which provides a preliminary starting point for the future investigation of several potential biomarkers associated with wound chronicity in humans.

## 1 Introduction

Chronic skin wounds (i.e., pressure ulcers, diabetic ulcers and vascular ulcers) exhibit a failure to restore barrier function due to impaired re‐epithelialization in response to injury. Wounds are diagnosed as impaired if they do not show a size reduction of at least 50% within 4 weeks of treatment [1]. The management of wounds and impaired cutaneous healing in humans is a multi‐faceted problem that has been growing worldwide along with the increasing diabetes and elderly populations. Canada alone spent over 12 billion dollars on wound care in 2023, exceeding previous estimates, while the United States led worldwide spending with an estimated cost of over 148 billion dollars in 2022 [2, 3, 4]. Despite these substantial investments in wound care, patients continue to suffer from pain, immobility and poor clinical outcomes. Current standards for chronic wound care focus on debridement of necrotic and non‐viable tissue, followed by frequent and laborious changes of compression dressings [1]. While many therapeutics have been shown to accelerate wound healing using in vitro experiments and animal models, no new therapies have shown reproducible success for human chronic wound treatment clinically in over 20 years [5]. The limited advancements in treatment within this field are likely a reflection of our incomplete understanding of the molecular pathology and aetiology of human chronic wounds.

Impaired cutaneous healing is associated with several comorbidities, but a consistent cell and molecular pathology underlying wound chronicity has not been clearly identified [6, 7, 8, 9, 10, 11, 12, 13, 14, 15, 16, 17, 18, 19, 20, 21]. However, a recent study comparing acute wounds to diabetic ulcers revealed that pro‐inflammatory cytokines and transcription factors necessary for the survival and function of immune cells present in acute wounds are reduced or absent in diabetic ulcers [22]. In addition to an altered inflammatory microenvironment, non‐healing skin wounds are also associated with localised hypoxia which can lead to increases in oxidative stress [18, 21, 23]. While reactive oxygen species (ROS) are essential for inducing keratinocyte migration, increased or prolonged oxidative stress has been associated with impaired wound healing [14, 16, 18, 21, 23, 24, 25]. Nuclear factor kappa‐light‐chain‐enhancer of activated B cells (NF‐κB) and nuclear translocation of nuclear factor erythroid 2‐related factor 2 (Nrf2) are transcription factors that regulate pro‐inflammatory and antioxidant gene expression respectively in multiple cell types including keratinocytes [26, 27]. Changes in both Nrf2 and NF‐κB expression have been assessed in response to therapeutics in human chronic wounds to infer modulation of either the antioxidant or inflammatory response respectively [28, 29]. However, their expression in keratinocytes of human chronic wounds is poorly characterised histologically, particularly with distance from wound edge (WE) [30]. Both Nrf2 and NF‐κB are indicated in contributing to re‐epithelialization through the upregulation of activation associated cytokeratin (CK) proteins; CK6, CK16 and CK17, necessary for hemidesmosome stabilisation and migration in the interfollicular epidermis [31, 32, 33]. Additionally, CK16 has been detected at the protein level at the leading edge of chronic wounds, while CK6 and CK17 have only been investigated at the mRNA level in chronic wounds showing increased expression at the leading edge [6, 20, 34].

The objective of the present study was to evaluate markers of keratinocyte activation, differentiation and oxidative stress, as potential biomarkers to guide debridement. We hypothesized that evidence of oxidative stress and keratinocyte activation would decrease as distance from the WE increases, and that endstage tissue will display an increase in oxidative damage than tissue from debridement. Using histology and immunohistochemistry (IHC) of human chronic wound debridement and endstage tissue we assessed: (1) skin architecture and composition, (2) keratinocyte activation and differentiation and (3) oxidative stress and damage, at the edge of the wounds and throughout the excised tissue.

## 2 Methods

### 2.1 Human Chronic Wound Samples

Collection of human tissue for analysis was conducted in accordance with the 1964 Declaration of Helsinki, and with approval from the University of Western Ontario Review Board for Health Sciences Research Involving Human Subjects. All participating patients provided written informed consent prior to study enrolment. Two groups of patients with non‐healing skin wounds were assessed; (1) patients that underwent elective amputation of the affected limb due to endstage critical limb ischemia (*n* = 10), and (2) patients that received routine surgical debridement of the wound for continued wound care (*n* = 6). Skin samples from amputation patients were collected from the wound area and from an area at least 10 cm from the WE, while skin samples from debridement patients only included the wound area. All skin samples were immediately fixed in the operating room using either 4% paraformaldehyde (Sigma‐Aldrich, St. Louis, MO) or 10% neutral buffered formalin (Sigma‐Aldrich). The demographics for each patient are listed in Table 1.

**TABLE 1:** Patient demographics.

Patient	Age	Sex	Diabetes status	Wound type
Amputation patients				
AP1	46	Female	Type 2 (poorly controlled)	Vascular ulcer
AP2	79	Female	Type 2 (insulin‐dependent)	Vascular ulcer
AP3	34	Male	Type 1	Diabetic foot ulcer
AP4	47	Male	Type 2 (insulin‐dependent)	Diabetic foot ulcer
AP5	55	Male	Type 2	Non‐healing stump
AP6	58	Male	Type 2	Diabetic foot ulcer
AP7	66	Male	Type 2	Diabetic foot ulcer
AP8	73	Male	Type 2 (insulin‐dependent)	Diabetic foot ulcer
AP9	79	Male	Type 2 (insulin‐dependent)	Diabetic foot ulcer
AP10	87	Female	Type 2 (insulin‐dependent)	Diabetic foot ulcer
Debridement patients				
AP11	31	Female	No	Trauma/pressure ulcer
AP12	51	Male	No	Trauma/pressure ulcer
AP13	61	Female	Type 2	Neuropathic foot ulcer
AP14	56	Male	No	Neuropathic foot ulcer
AP15	74	Male	N/A	Neuropathic foot ulcer
AP16	51	Male	Type 2	Neuropathic foot ulcer
AP17	77	Male	No	Neuropathic foot ulcer
AP18	62	Female	No	Pressure ulcer (Heel)

### 2.2 Histological Analysis and IHC

Histology, IHC and imaging were conducted as previously described [35]. Specific antibody information and parameters of use are listed in Table 2. Staining was also conducted without primary antibodies to confirm true staining and rule out non‐specific secondary antibody binding (Figure S1).

**TABLE 2:** List of antibodies and associated information.

Primary antibody specificity	Vendor	Catalogue number	Assay conc.	Antigen retrieval	Secondary antibody
CK14	Abcam	ab7800	1:100	Yes	AF 647 Dn anti‐mouse
CK10	Abcam	ab76318	1:200	Yes	AF 555 Dn anti‐rabbit
ECAD	BD Biosciences	610,181	1:500	Yes	AF 555 Dn anti‐mouse
FLG	Abcam	ab81468	1:200	Yes	AF 647 Dn anti‐rabbit
CK6	Abcam	ab18586	1:100	Yes	AF 647 Dn anti‐mouse
CK16	Thermo Fisher Scientific	PA5‐109897	1:200	Yes	AF 647 Dn anti‐rabbit
CK17	Abcam	ab53707	1:400	Yes	AF 555 Dn anti‐rabbit
PCNA	Santa Cruz Biotechnology	sc‐56	1:250	Yes	AF 647 Dn anti‐mouse
CD4	Thermo Fisher Scientific	14‐2444‐82	1:200	Yes	HRP‐DAB
p‐NF‐κB	Abcam	ab86299	1:200	Yes	AF 647 Dn anti‐rabbit
p‐Nrf2	Abcam	ab76026	1:200	Yes	AF 647 Dn anti‐rabbit
8‐OHdG	Abcam	ab48508	1:200	Yes	AF 647 Dn anti‐mouse
3‐NT	Abcam	ab61392	1:500	No	AF 647 Dn anti‐mouse
4‐HNE	Abcam	ab46545	1:500	No	AF 555 Dn anti‐rabbit

### 2.3 Image Analysis and Quantification

QuPath software [36] was used for all image analysis, quantification and isolation of regions of interest for figures. Raw .czi images generated using Zen imaging software were organised into QuPath projects for comparison and analysis to ensure no data were lost to file compression. Tissue from debridement patients was divided into two regions for analysis, WE and adjacent unwounded (ADJ, 3–10 mm from the WE), while tissue from endstage patients included a third region for analysis, non‐involved (NI) skin from the amputated limb collected from at least 10 cm away from the wound. Quantification of nuclear localization in the epidermis for phosphorylated (p‐) NF‐κB (Figure 5I), p‐Nrf2 (Figure 6I) and 8‐hydroxy‐2′‐deoxyguonosine (8‐OHdG) (Figure 7I) was done as previously described [35].

### 2.4 Statistical Methods

Statistical analysis was conducted using GraphPad Prism v5 software (*p* ≤ 0.05 was considered significant). Normality was assessed with the Kolmogorov–Smirnov test and data were found to be non‐parametric. Nuclear localization was compared between all regions of interest and time‐points for p‐NF‐κB (Figure 5I), p‐Nrf2 (Figure 6I) and 8‐OHdG (Figure 7I), using the Kruskal–Wallis test with Dunn's multiple comparison test. Data are presented as mean value for each measurement with significance set at *p* < 0.05, and each significantly different comparison was marked with an asterisk (*).

## 3 Results

### 3.1 Phenotypes of the Epidermis Associated With Impaired Re‐Epithelialization of Chronic Wounds

**FIGURE 1:**
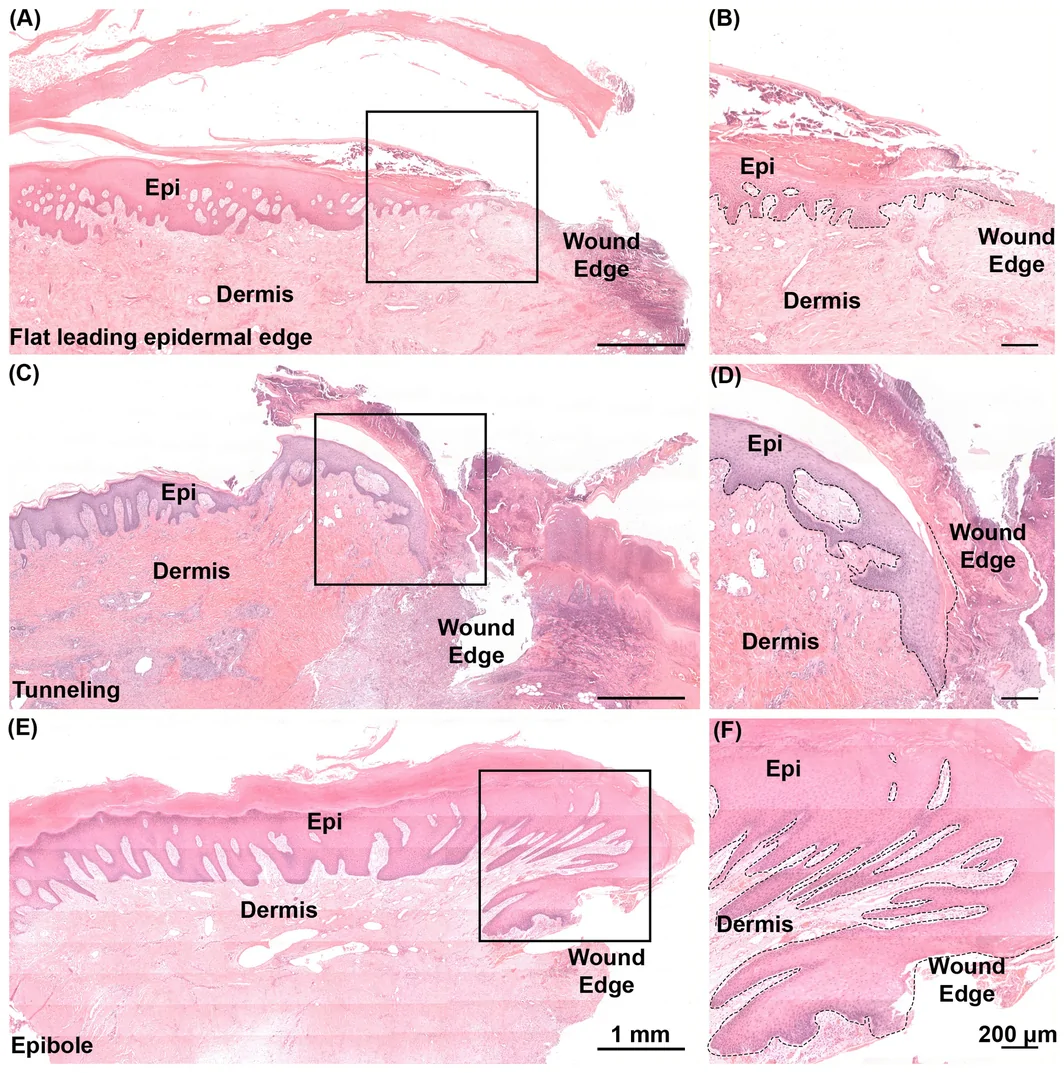
Representative histology of the wound edge phenotypes of human chronic wounds. (A) Representative example of a wound with a flat leading epidermal edge on the surface of the wound bed with an inset of the wound edge epidermis (B). (C) Representative example of a wound with the leading epidermal edge tunnelling into the wound bed with an inset of the wound edge epidermis (D). (E) Representative example of a wound displaying epibole at the leading epidermal edge with an inset of the wound edge epidermis (F). Scale bars are 1 mm (A, C, E) and 200 μm (B, D, F). Epi = epidermis. Dashed lines separate the dermis and epidermis (B, D, F).

The phenotypes of each wound were assessed focussing on hyperproliferation, hyperkeratosis and the directionality of the epidermis at the leading edge (Table 3). Hyperproliferation of the epidermis was detected in 9/10 endstage tissue but was primarily evident in the epidermis adjacent to the wound while the WE epidermis reduced in thickness in 8/10 endstage tissues. In debridement wounds however, hyperproliferation of the epidermis was evident in 7/8 samples at the WE and 5/8 samples adjacent to the wound. Hyperkeratosis was also evident in the majority of both endstage and debridement tissue primarily at the WE and often extending to the adjacent epidermis, only being absent in 2/10 endstage and 2/8 debridement tissue. Additionally, abnormal retention of nuclei, parakeratosis, was detected in the stratum corneum of 8/10 endstage and 5/6 debridement tissue. In both endstage and debridement tissue, we identified three phenotypes associated with the orientation of the WE epidermis. In the first phenotype, the epidermis laid flat on the surface of the wound bed (Figure 1A,B), evident in 6/10 endstage and 2/8 debridement samples (Table 3). In the second phenotype the leading edge of the epidermis appears to have achieved some degree of migration across the wound bed while tunnelling beneath the surface (Figure 1C,D). This tunnelling phenotype was identified in 2/10 endstage and 2/8 debridement samples (Table 3). The third phenotype observed displayed similar tunnelling of the epidermis to the second phenotype; however, instead of tunnelling across the wound bed, the leading edge of the epidermis tunnelled down and back under itself away from the wound bed, a phenomenon commonly referred to as epibole (Figure 1E,F). Epibole was identified in 2/10 endstage and 4/8 debridement samples (Table 3).

**TABLE 3:** Wound phenotypes.

Patient	Hyperproliferation	Hyperkeratosis and parakeratosis	WE orientation
Amputation patients			
AP1	ADJ	WE with parakeratosis	Flat on WB surface
AP2	ADJ	WE with parakeratosis	Flat on WB surface
AP3	ADJ and WE	ADJ and WE with parakeratosis at WE only	Tunnelling with epibole
AP4	ADJ	Hyperkeratosis only at WE	Tunnelling in WB
AP5	No	No	Flat on WB surface
AP6	ADJ	ADJ and WE with parakeratosis	Flat on WB surface
AP7	ADJ	Parakeratosis only at WE	Flat on WB surface
AP8	ADJ	ADJ and WE with parakeratosis at WE only	Tunnelling in WB
AP9	ADJ	ADJ and WE with parakeratosis	Flat on WB surface
AP10	ADJ and WE	ADJ and WE with parakeratosis	Tunnelling with epibole
Debridement patients			
AP11	ADJ and WE	ADJ and WE with parakeratosis at WE only	Tunnelling in WB
AP12	WE	WE only with parakeratosis	Tunnelling with epibole
AP13	ADJ and WE	ADJ and WE with parakeratosis at WE only	Tunnelling with epibole
AP14	ADJ and WE	ADJ and WE with severe parakeratosis	Tunnelling
AP15	No	No	Flat on WB surface
AP16	WE	No	Flat on WB surface
AP17	ADJ and WE	ADJ and WE with parakeratosis at WE only	Tunnelling with epibole
AP18	ADJ and WE	ADJ and WE with parakeratosis at WE only	Tunnelling with epibole

Abbreviations: ADJ: adjacent, WB: wound bed, WE: wound edge.

### 3.2 Expression of CK14 and CK10

**FIGURE 2:**
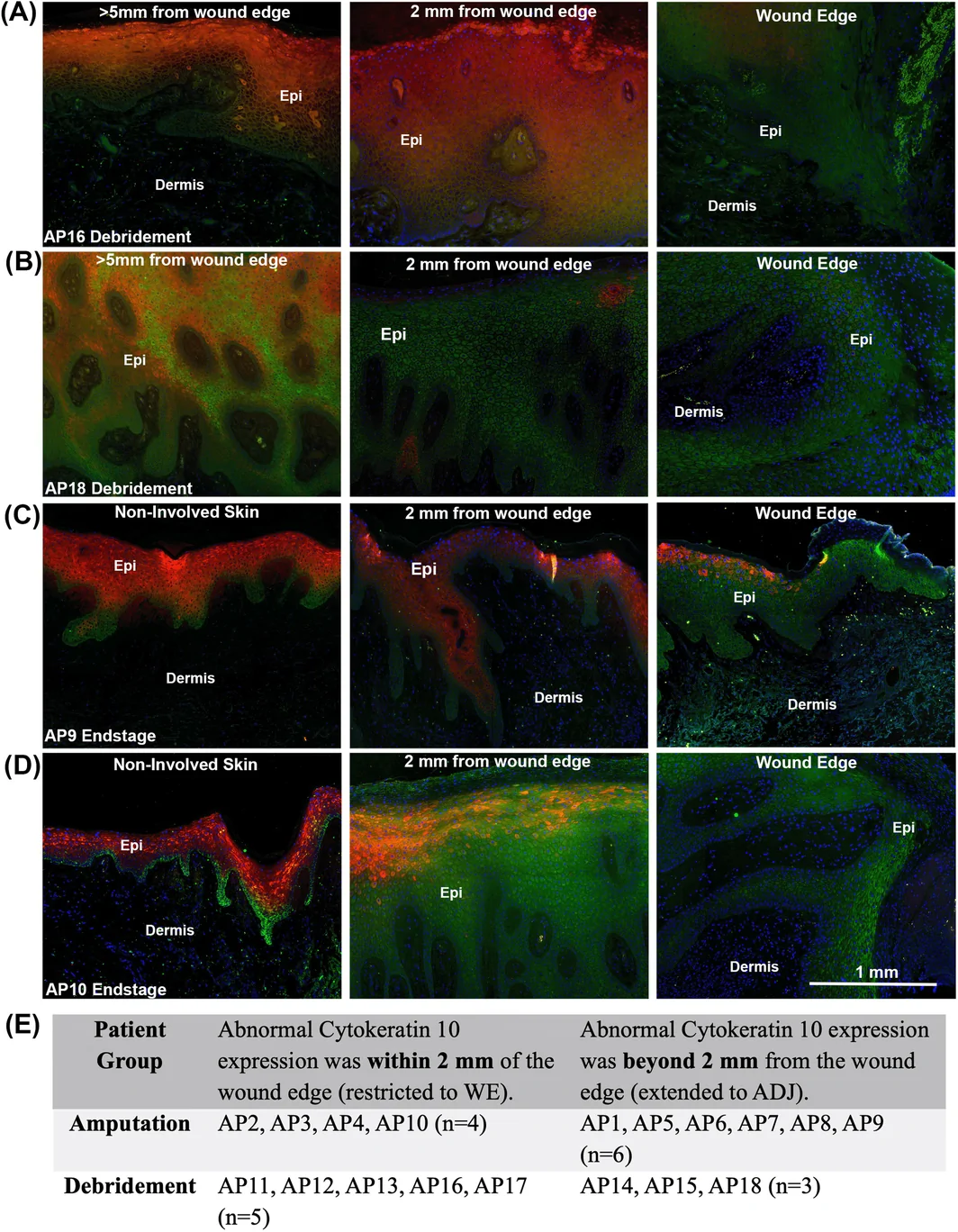
Evaluation of keratinocyte differentiation phenotypes through the expression of basal associated CK14 and suprabasal associated CK10. Representative images of CK10 and CK14 localization in debridement (A, B) and endstage tissue samples (C, D). Samples were assessed at > 5 mm from wound edge, 2 mm from wound edge and the wound edge epithelium (E) Table grouping patients by CK10 expression phenotype and localization surrounding the wound. Scale bars are 1 mm (A, C, E) and 200 μm (B, D). Epi = epidermis.

Basal keratinocyte associated CK14, and suprabasal keratinocyte associated CK10 were visualised using co‐fluorescent IHC to assess the differentiation status of keratinocytes associated with impaired healing. In the control samples used in the study, NI epidermis of the endstage wounds, CK14 expression was observed only in the basal layer of keratinocytes, and CK10 expression was detected throughout the suprabasal keratinocyte layers of the epidermis (Figure 2A–D). In the WE and adjacent epidermis of both endstage and debridement samples CK14 expression was observed throughout the basal and suprabasal keratinocyte layers in the WE tissue, while CK10 was reduced displaying a spectrum of expression patterns and spatiolocalisation. (Figure 2A–D). In a subset of debridement and endstage tissues, CK10 expression was absent in the WE epidermis but was present in suprabasal keratinocyte layers within 2 mm of the WE, with a transitional region displaying a mosaic pattern of CK14 and CK10 expression in the epidermis and normal CK10 expression throughout the suprabasal layers of the adjacent epidermis (Figure 2B,D). This mosaic pattern of CK10 was present in 5/8 debridement samples and 4/10 endstage tissues (Figure 2E).

### 3.3 E‐Cadherin and Filaggrin Expression

**FIGURE 3:**
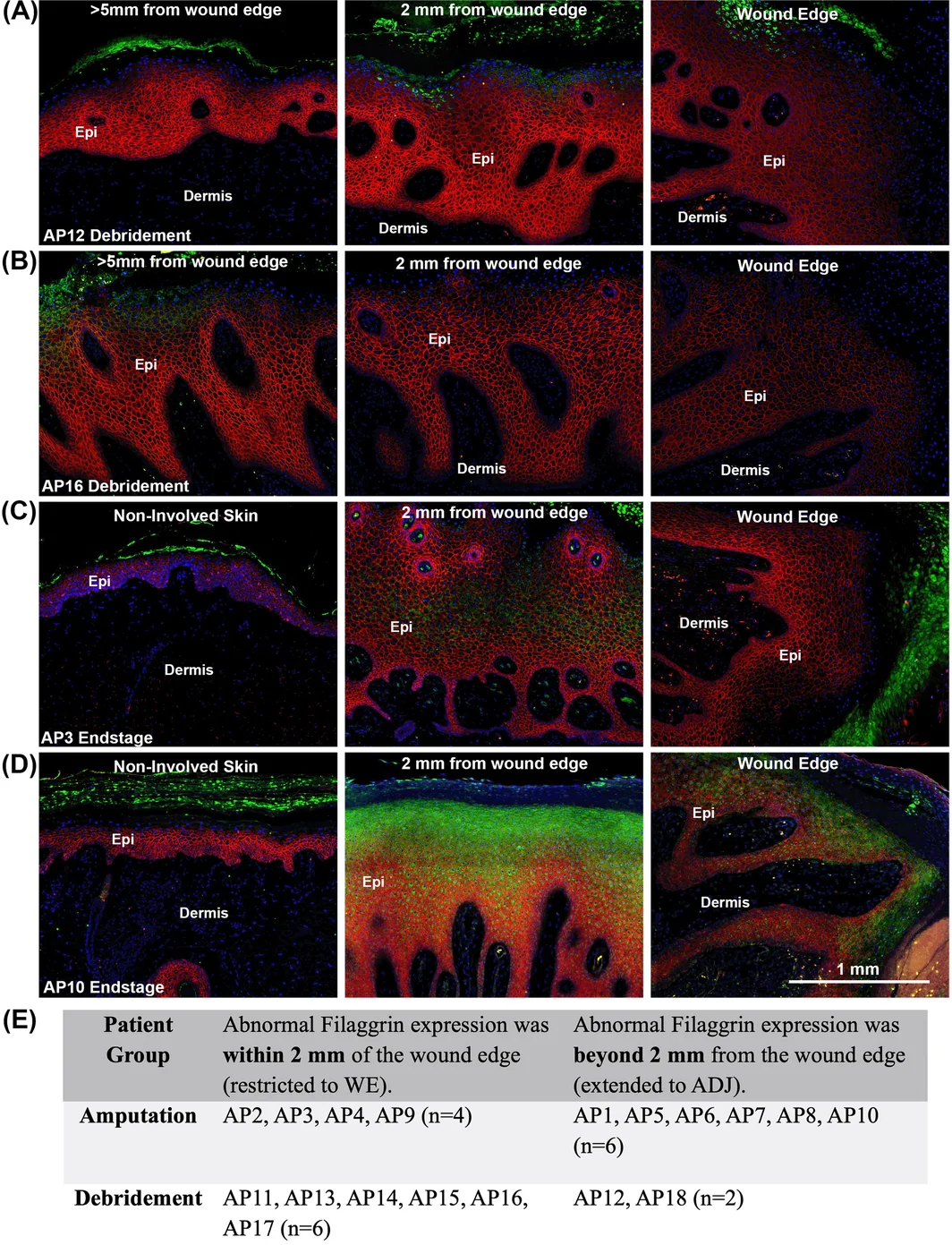
Assessment of cell–cell adhesion, barrier function and terminal differentiation of keratinocytes through E‐cadherin and filaggrin expression. Representative images of E‐cadherin and filaggrin localization in debridement (A, B) and endstage tissue samples (C, D). Samples were assessed at > 5 mm from wound edge, 2 mm from wound edge and the wound edge epithelium (E) Table grouping patients by filaggrin expression phenotype. Scale bar is 1 mm (A). Epi = epidermis. Dashed lines separate the dermis and epidermis.

E‐cadherin was expressed and localised to the borders of keratinocytes throughout the NI epidermis while filaggrin expression was detected throughout the stratum corneum and within the intracellular granules of the stratum granulosum (Figure 3A–D). ECAD expression was consistent through the epidermis 2 mm from WE, but both endstage and debridement samples displayed reduced density of E‐cadherin expression in the WE epidermis, primarily in the most suprabasal layers (Figure 3A–D). Filaggrin expression showed more variability in both endstage and debridement samples (Figure 3A–D). In a subset of tissues from each group, filaggrin was expressed in the stratum corneum and granulosum in the adjacent epidermis and into the WE epidermis within 2 mm of the leading edge (Figure 3A,C,D). In other debridement and endstage samples filaggrin expression was absent in the stratum corneum of the WE epidermis extending beyond 2 mm from the WE into the adjacent epidermis, however expression of filaggrin can be observed intracellularly in the epidermis (Figure 3B). While all samples exhibited an abnormal localisation of filaggrin, it only persisted beyond 2 mm of the WE in 2/8 debridement samples and 6/10 endstage tissues (Figure 2E).

### 3.4 Expression of Keratinocyte Activation Associated With CK6, CK16 and CK17

**FIGURE 4:**
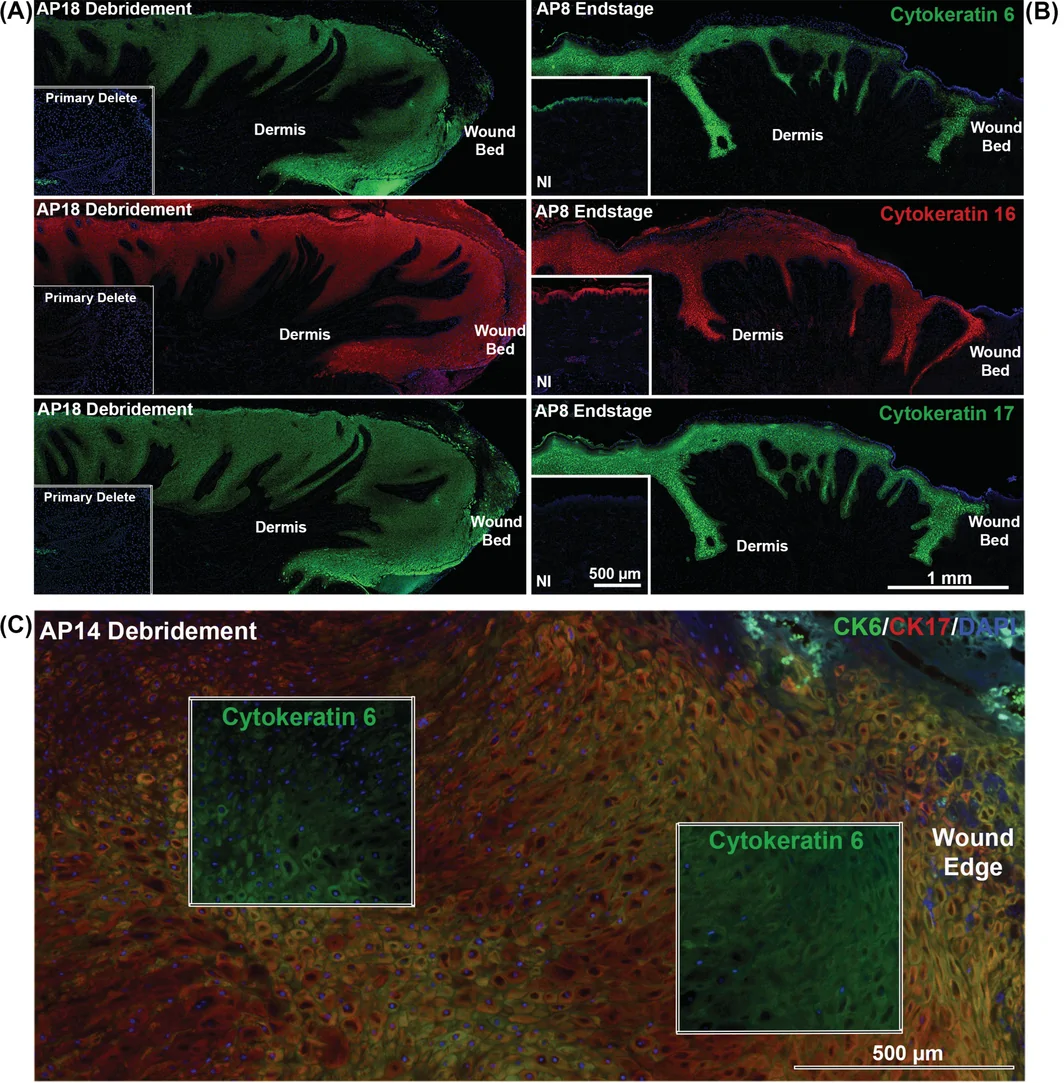
Assessment of keratinocyte activation through expression of CK6, CK16 and CK17 at the wound edge. Representative images from wounds at debridement (A) and endstage at amputation (B). Co‐localization of CK6 and CK17 in debridement wound edge tissue (C).

To assess keratinocyte activation at the WE, we used antibodies specific to CK6, CK16 and CK17. CK6 expression was detected through the suprabasal layers of the WE epidermis and into the adjacent epidermis of endstage and debridement samples (Figure 4A,B). Expression of CK16 and CK17, however, were both detected in abundance in the WE epidermis and extended through the adjacent and NI epidermis of all endstage samples and suprabasally in the WE and adjacent epidermis of debridement samples (Figure A, B, C). In samples exhibiting extensive hyperproliferation at the WE, both CK6 and CK17 were highly expressed (Figure 4C).

### 3.5 NF‐κB and Nrf2 Expression, Phosphorylation and Nuclear Translocation in Keratinocytes

**FIGURE 5:**
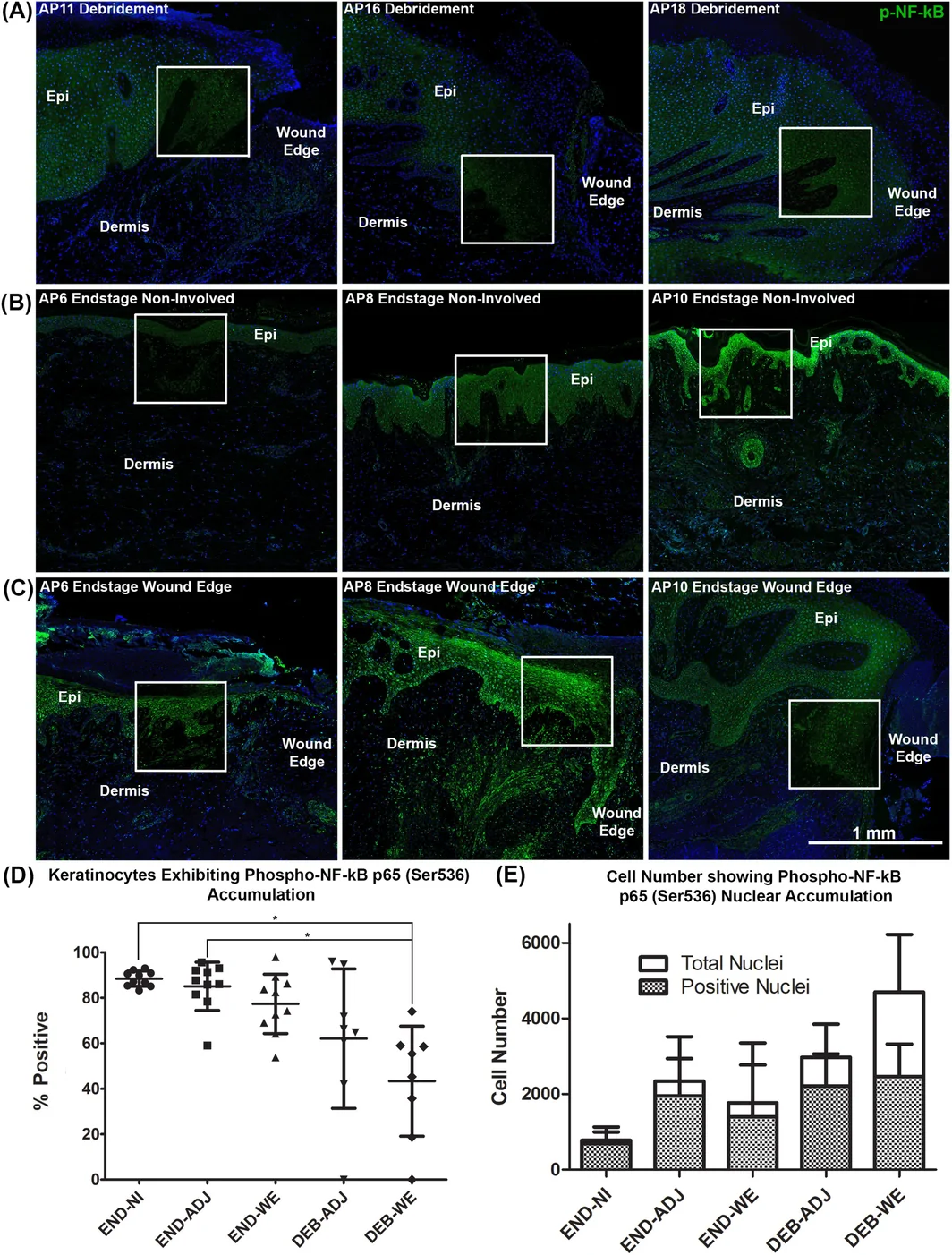
Localization of NF‐κB p65 (phospho ser536) activation in wound edge and adjacent tissue. Representative images of NF‐κB p65 (phospho ser536) localization in debridement wound tissue (A), endstage non‐involved skin (B) and endstage wound edge samples (C). Insets show NF‐κB p65 (phospho ser536) labeling without DAPI channel. Quantification of cells showing NF‐κB p65 (phospho ser536) activation within the epidermis, represented as percent positive cells (D) and cell number (E). * represents significant differences between groups where *p* = 0.05.

**FIGURE 6:**
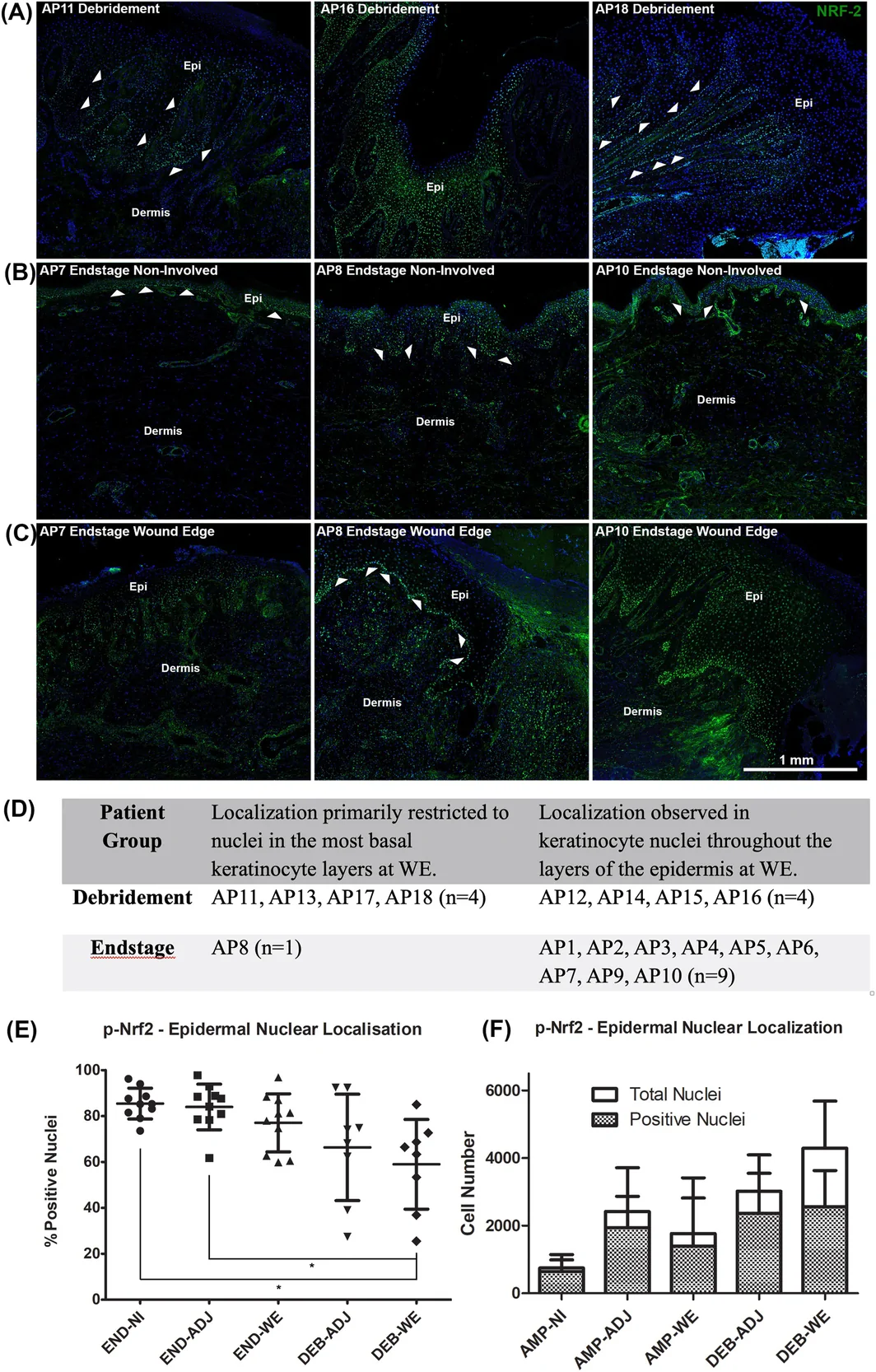
Evaluation of antioxidant transcription factor Nrf2 (phospho ser40) through protein expression, activation via phosphorylation and nuclear localisation. Representative images of Nrf2 (phospho ser40) localisation in debridement wound tissue (A), endstage non‐involved skin (B) and endstage wound edge samples (C). Assessment of pattern of localisation in each sample subdivided into basal localisation versus through the entire thickness of epidermis (D). Quantification of nuclear localisation within the epidermis, represented as (E) percent positive cells and (F) cell number. * represents significant differences between groups where *p* = 0.05.

The phosphorylation and nuclear translocation of NF‐κB and Nrf2 were used to evaluate the activation of pro‐inflammatory and antioxidant signalling pathways respectively. In debridement and endstage samples, both p‐NF‐κB and p‐Nrf2 were detected in WE tissue (Figures 5A–C and 6A–C). Two specific patterns of p‐Nrf2 localization were observed: restricted to basal keratinocytes or present throughout all layers of the WE epidermis (Figure 6A–D). Compared with WE in endstage wounds, samples at debridement showed significantly fewer p‐NF‐κB and p‐Nrf2 positive nuclei detected (Figures 5A,D,E and 6A,E,F). In endstage samples, both phosphorylated (p‐) NF‐κB and p‐Nrf2 were highly detected within nuclei of the NI, adjacent and WE epidermis (Figures 5A–C and 6A–C) with no significant differences between the regions for either transcription factor (Figures 5D,E and 6E,F).

### 3.6 Accumulation of Oxidative Damage to DNA, Proteins and Lipids

**FIGURE 7:**
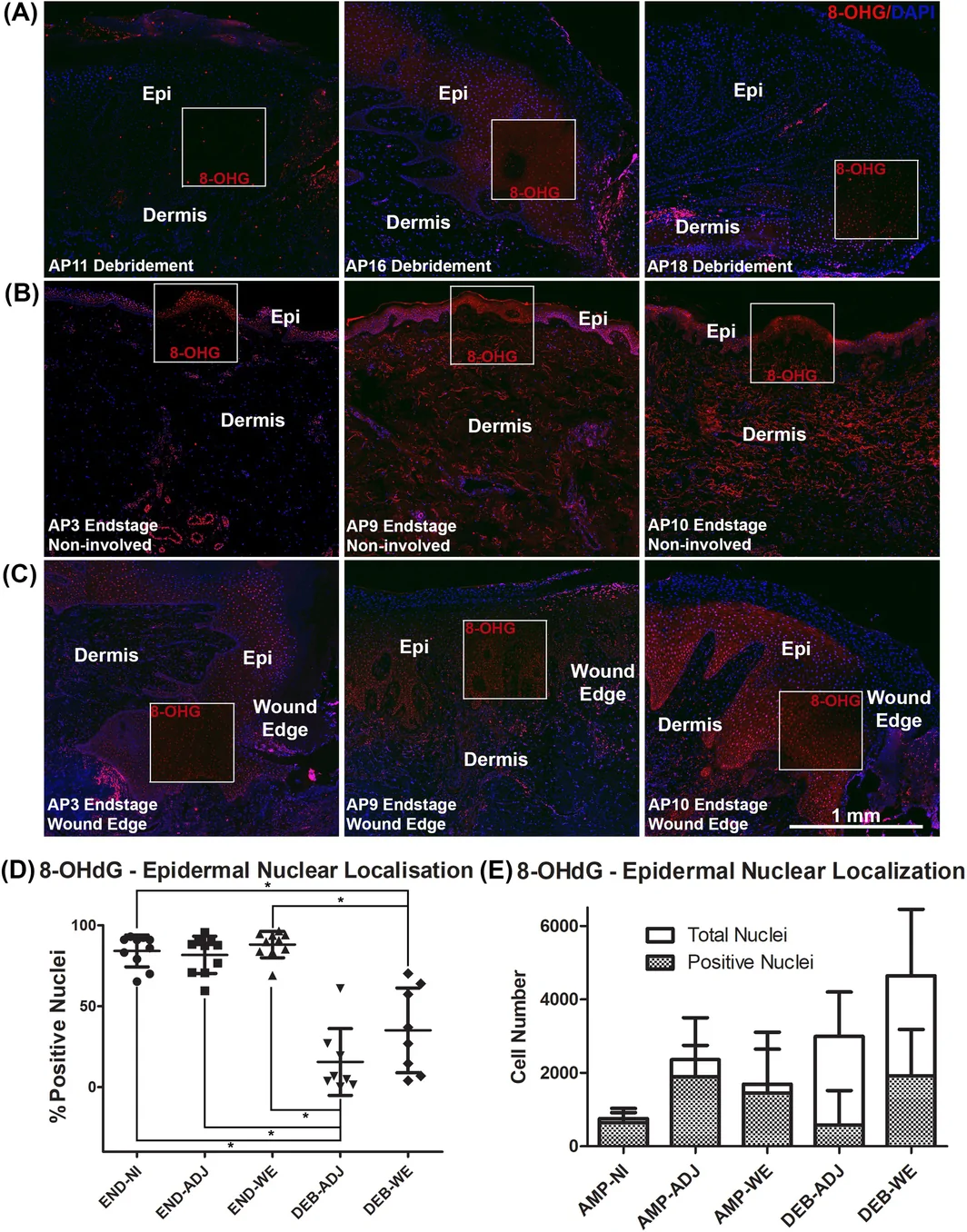
Assessment of oxidative DNA damage through the nuclear accumulation of 8‐OHdG. Representative images of 8‐OHdG localization in debridement wound tissue (A), endstage non‐involved skin (B) and endstage wound edge samples (C). Insets show 8‐OHdG labeling without DAPI channel. Quantification of nuclear localization within the epidermis, represented as percent positive cells (D) and cell number (E). * represents significant differences between groups where *p* = 0.05.

DNA damage resulting from oxidative stress was measured using fluorescent IHC for 8‐OHdG and oxidative damage to proteins and lipids was assessed through fluorescent IHC for 3‐nitrotyrosine (3‐NT) and 4‐hydroxynoneal (4‐HNE) respectively. 8‐OHdG, indicative of guanosine oxidation, showed variable nuclear translocation in debridement samples, with certain samples exhibiting none and other wounds showing nuclear translocation throughout the WE epidermis (Figure 7A). In wound samples at endstage, nuclear translocation of 8‐OHdG showed nuclear localization throughout all layers of the epidermis at the WE, adjacent to the wound, as well as in NI skin (Figure 7B,C), with no differences in detection observed between these different regions (Figure 7D,E).

**FIGURE 8:**
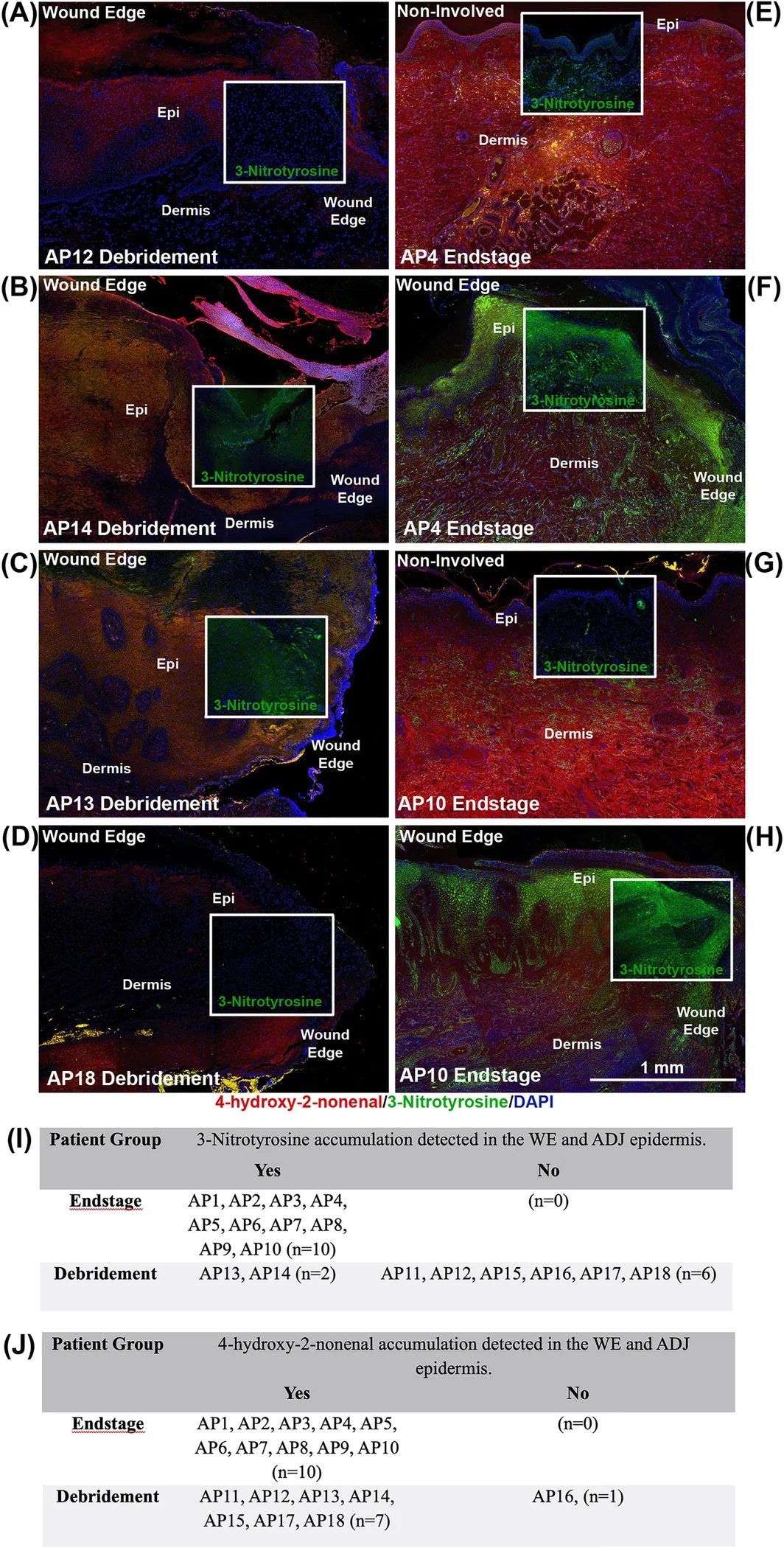
Analysis of oxidative damage to lipids and proteins through the accumulation of lipid peroxidation marker 4‐HNE, and protein nitration marker 3‐NT respectively. Representative images of 3‐NT and 4‐HNE localization in debridement wound tissue (A, B, C, D), endstage non‐involved skin (E, G) and endstage wound edge samples (F, H). Table grouping patients based on the presence or absence of 3‐NT (I) and 4‐HNE (J) accumulation in the WE and ADJ epidermis.

To assess protein damage and lipid peroxidation, antibodies specific to 3‐NT and 4‐HNE were applied to the WE and adjacent epidermis of all debridement and endstage samples (Figure 8). In debridement samples, 4‐HNE was detected within the WE epidermis with more variation present in adjacent skin (Figure 8A–D,J). 3‐NT was present in only two debridement samples at the WE (Figure 8B,C) and was absent in all other debridement samples examined (Figure 8A,D,I). In endstage samples, both 3‐NT and 4‐HNE were present in WE epidermis (Figure 8E–G), although epidermal localization was lower in NI skin (Figure 8E,G).

## 4 Discussion

Impaired cutaneous wound healing places a massive burden on both patients and healthcare systems globally [2, 3, 4]. Characterised by reduced re‐epithelialization, dysregulated inflammation, and increased oxidative stress, there are many significant knowledge gaps on the molecular pathophysiology that limit chronic wound treatment and development of new therapeutics [6, 14, 18, 21, 23, 24, 25]. The objective of this study was to compare chronic skin wound tissue isolated from routine debridement procedures with that from endstage wounds recovered at limb amputation for markers associated with oxidative stress, keratinocyte activation and keratinocyte differentiation. By improving our understanding of the aetiology of chronic wounds and impaired re‐epithelialization, important insights can be gained to guide physicians in optimising debridement procedures and researchers in the development of effective therapeutic options to promote re‐epithelialization. Further, by making comparisons between debridement tissue and those at endstage, it is conceivable to identify markers that could be used to inform clinical decisions including need for skin grafting or when amputation becomes a necessity.

Re‐epithelialization as a process involves the proliferation and migration of keratinocytes in direct apposition to the wound [13, 15, 16, 37, 38] which is associated with keratinocyte activation; WE keratinocytes change expression of CKs which is considered to alter cell stiffness to promote migration [12, 16, 33]. We observed hyperproliferation and hyperkeratosis in the adjacent epidermis and epithelial tongue in many debridement samples; but in contrast, in most endstage samples, hyperproliferation was more common in epidermis adjacent to the wound but not at the WE itself. Interestingly, several debridement samples exhibited epibole (rolled edges), but the majority of endstage tissues displayed a thinner epidermis with very few stratified layers. These observations show that the epidermis in debridement samples still shows proliferative and migratory capacity but is lacking the appropriate guidance cues necessary to direct migration across the wound bed. Alternatively, the microenvironment provided by the WE dermis is preferable to that of the wound bed which is known to be pro‐inflammatory [6, 7, 14, 39, 40]. In contrast, in endstage tissue, the potential of the WE epidermis to proliferate and migrate is clearly attenuated, which could be linked to critical limb ischemia and persistent hypoxia in the limbs. In summary, our data shows that epithelial dynamics and morphology vary in wound chronicity and we next investigated whether this could be linked specifically to the keratinocyte activation cycle and differentiation state.

In quiescent skin, basal keratinocytes are distinguished through expression of CK14; CK10 is associated with suprabasal, differentiating keratinocytes and filaggrin associated with the most suprabasal and most differentiated keratinocytes [12, 13, 41, 42]. Interestingly, both debridement and endstage samples show expression of CK14 throughout the suprabasal keratinocyte layers in all samples, although variability was observed in the expression of CK10, particularly with distance from WE. In a subset of debridement samples, CK10 expression was evident within 2 mm of the WE but was present in certain cell populations showing a mosaic‐like pattern. In contrast, most endstage tissue samples showed an absence of CK10 expression that extended into epidermis more than 2 mm away from the WE (Figure 2). The expression of filaggrin followed the same pattern of expression as CK10 in all patients (Figure 3). Our data is in general agreement with acute human excisional wounds [34]. Other studies comparing acute wounds at 7 days post wounding to chronic diabetic ulcers showed similar CK14 expression increased in the suprabasal keratinocyte layers at the WE and CK10 expression attenuated but still expressed in the most suprabasal layers at the WE [20]. The expression of CK14 in suprabasal cells has been suggested to provide evidence for the leapfrog hypothesis of epithelial migration [13, 20, 34]. Specifically, it has been hypothesized that suprabasal cells ‘dedifferentiate’, or that basal cells undergo altered differentiation, retaining CK14 and downregulating CK10 expression, to provide cells for the epithelial tongue [35]. In summary, our data suggest that in chronic wounds at the point of debridement, the WE epidermis exhibits a similar differentiation phenotype to acute wounds. However, in chronic wounds associated with endstage ischemia and amputation, this altered differentiation or ‘dedifferentiation’ phenotype extends far into the uninjured epidermis. This suggests that molecular signals provided by both the wound bed and intact dermis could be similar, exacerbating epidermal dysfunction. We next assessed if the differences in epidermal differentiation states could be linked to stress‐induced keratinocyte activation.

Associated with the keratinocyte activation cycle, we show that CK6, CK16 and CK17 expression is present through the suprabasal epidermal layers in debridement samples extending from the WE into surrounding uninjured skin. In endstage wounds, CK6 was typically expressed suprabasally in the WE and adjacent epidermis, but CK16 and CK17 were expressed in all layers of the epidermis at the WE, in adjacent uninjured skin, as well as in NI skin, again implicating limb ischemia in regulation of CKs and basal keratinocyte expression of CK16 and CK17 (Figure 4). Studies in acute human wounds demonstrated the expression of CK6, CK16 and CK17 throughout the WE and the epithelial tongue during reepithelialization (day 0 to day 6 post wounding), persisting even after completion of re‐epithelialization. However, their expression was never detected in the uninjured, adjacent interfollicular epidermis [10, 13]. These data further suggest that the uninjured adjacent and NI epidermis in chronic wound tissue are receiving signals that in acute wounds are only present in the WE epidermis, which could impact on cellular rigidity, desmosome destabilisation, hyperproliferation and migration normally associated with CK6, CK16 and CK17 [33], potentially weakening the epidermis surrounding the wound and reducing growth factors and other signalling molecules required at the WE. With respect to human wound chronicity, CK16 protein expression has been previously described in the same spatial pattern evident in acute wounds [20]. However, CK6 and CK17 expression has only been detected through RNA analysis at the leading edge of chronic wounds and their spatial or cellular localization is unknown [6]. As the expression of CK6, CK16 and CK17 in debridement and endstage WE keratinocytes is similar to that evident in acute wounds, it demonstrates that the upregulation of these stress induced CKs is likely not the reason for wound chronicity. Moreover, it highlights that when wounds show impaired healing, the expected underlying CK gene and protein expression still occur showing that the response is in part ‘normal’ even at endstage.

Wound chronicity is strongly associated with increased oxidative stress [18]. Accumulation of ROS, which while required for keratinocyte activation, can cause tissue damage if it remains chronically elevated [16, 18, 21, 23, 24, 25]. The regulation of ROS in keratinocytes is largely orchestrated through the pro‐inflammatory transcription factor NF‐κB and the antioxidant transcription factor Nrf2, which are also both associated with the upregulation of CK6, CK16 and CK17 [26, 27, 43]. Our analysis of p‐NF‐κB and p‐Nrf2 nuclear localization in keratinocytes revealed consistent overlap of the transcription factors in both debrided and endstage tissue (Figures 5 and 6). Of great significance, we observed significantly increased nuclear translocation of both transcription factors in the adjacent uninjured and NI epidermis of endstage samples compared to the WE epidermis of debridement tissues, where restriction to basal keratinocytes was evident in certain samples (Figures 5 and 6). These observations suggest the severity of chronic wounds and limb ischemia is associated with an increased inflammatory response in the epidermis beyond the WE. Nrf2 and NF‐κB play inhibitory roles in each other's function through competition for transcription cofactors and DNA binding proteins, as well as through promoting the function of inhibitory molecules in each other's signalling pathways [26, 27]. Therefore, the activation and nuclear localization of both transcription factors together could suggest impaired function of one or both signalling cascades or a maladaptive response.

Further evidence for a maladaptive response being associated with wound chronicity is the oxidative damage to DNA, proteins and lipids in the epidermis, suggesting that the activation of Nrf2 observed is insufficient to mitigate the oxidative stress particularly in endstage wounds. Interestingly, in debridement samples, Nrf2 was observed to localise to basal keratinocytes only in 50% of the wounds (vs. 10% in endstage), which raises the question of which factors influence its activation. The type of damage evident in the tissue is predominantly DNA damage and lipid peroxidation, likely due to increased ROS production in the tissue. The extensive expression and activation of Nrf2 and NF‐κB observed in endstage wounds suggests that previously theorised therapeutic interventions aimed at increasing Nrf2 expression [28, 29, 30, 44, 45, 46] may have limited effectiveness after a certain point in chronic wound progression and direct stimulation or supplementation of its downstream antioxidant effector proteins could prove more useful.

Interestingly, in debridement samples, protein nitrosylation was absent in six of the eight samples, but this is an active regulated post translational modification [47]. It requires specific enzymes such as transnitrosylases which actively transfer NO groups to cysteine targets [48]. In contrast, lipid peroxidation and guanosine oxidation are reactions in which free radicals (ROS) and oxidants remove electrons from lipids or guanosine respectively. All amputation‐derived wound samples show protein nitrosylation, suggesting that there is a change in the molecular aetiology of wound chronicity over time or that limbs ischemia results in more active nitrosylation. It does open the possibility of protein nitrosylation as a potential biomarker of wound progression. Although it would need to be confirmed in a higher number of wounds.

While informative on chronic wound pathophysiology, there are limitations that need to be considered. For this study, the cohort of patients we had access to was limited in number and restricted to a single geographic region. Debridement samples with epidermis are extremely limited in availability as clinicians rarely disturb epithelial tongues unless edges are rolled or exhibiting epibole. Additionally, this study was limited to examining wounds using histology of formalin fixed, paraffin embedded tissue to investigate protein expression and localization while the addition of RNA analysis in future studies could provide valuable insight on transcription factor function and whether the absence of key proteins is based on deficits in gene transcription or protein translation. Finally, the analysis in this study is based on a single tissue sample from each individual. Following patients longitudinally using non‐invasive methods such as wound exudate analysis along with the collection of debridement tissue could better elucidate wound progression, especially when aligned with clinical endpoint.

To conclude, this study has made previously undescribed comparisons between chronic wounds retrieved at routine debridement versus wounds sampled at amputation. Through these comparisons we have identified differences regarding the morphology of the epidermis at the WE, expression of CKs and intermediate filament associated proteins, activation of pro‐inflammatory and antioxidant transcription factors, and accumulation of oxidative damage. While preliminary, these differences provide multiple potential starting points for further investigation focused on identifying biomarkers to guide debridement and amputation protocols, as well as therapeutic intervention in wound chronicity.

## Author Contributions

D.K. and P.C. performed tissue harvest from debridements in their clinic and provided important guidance on clinical parameters. D.T. and J.C. processed the tissue samples, as well as staining, imaging and data analysis. D.W.H. and D.T. prepared the figures. D.W.H. designed the research study. D.T., D.K., P.C., J.C. and D.W.H. wrote and edited the manuscript.

## Funding

This work was supported by Canadian Institutes of Health Research, Grant no: 247506 to DWH and DK.

## Conflicts of Interest

The authors declare no conflicts of interest.

## Supporting information


**Figure S1:** Validation of IHC staining through comparison with tissue matched primary antibody controls. (A) Endstage wound tissue from patient F87 stained for CK10 and CK14 with fluorescent co‐IHC using AF647 anti‐Rb and AF555 anti‐Ms secondary antibodies. (B) Endstage wound tissue from patient F87. (C) Debrided wound tissue from patient M51 incubated with antibodies to filaggrin and E‐cadherin with fluorescent co‐IHC using AF647 anti‐Rb and AF555 anti‐Ms secondary antibodies. (D) Debrided wound tissue from patient M51 incubated with AF647 anti‐Rb and AF555 anti‐Ms secondary antibodies. (E) Endstage wound tissue from patient F87 stained for p‐Nrf2 with fluorescent IHC using AF647 anti‐Rb secondary antibodies. (F) Endstage wound tissue from patient F87 incubated with AF647 anti‐Rb secondary antibodies. (G) Endstage wound tissue from patient F31 stained for NF‐kB incubated with AF647 anti‐Rb secondary antibodies. (H) Endstage wound tissue from patient F31 incubated with AF647 anti‐Rb secondary antibodies. (I) Debrided wound tissue from patient F31 stained for 3‐NT and 4‐HNE with fluorescent co‐IHC using AF647 anti‐Ms and AF555 anti‐Rb secondary antibodies. (J) Debrided wound tissue from outlier patient F61 incubated with AF647 anti‐Ms and AF555 anti‐Rb secondary antibodies, without primary antibodies.

## Data Availability

The data that support the findings of this study are available on request from the corresponding author. The data are not publicly available due to privacy or ethical restrictions.
